# Health outcome convergence in Africa: the roles of immunization and public health spending

**DOI:** 10.1186/s13561-023-00436-9

**Published:** 2023-05-10

**Authors:** Ariane Ephemia Ndzignat Mouteyica, Nicholas Ngepah

**Affiliations:** School of Economics, University of Johannesburg, Johannesburg, South Africa

**Keywords:** Convergence, Public health spending, Immunization, Convergence clubs, Log t-test

## Abstract

**Background:**

Reducing health outcomes disparities in Africa is a major concern for policymakers. Inter-country disparities in Africa is well documented. However, little is known about the accurate trajectory of these disparities over time. Thus, this paper investigates the convergence hypothesis in health outcomes in 40 African countries using data from the World Development Indicators. The study used panel data from 2000 to 2019.

**Method:**

The study employs a nonlinear time-varying factor model to test the convergence hypothesis on infant mortality rate, under-five mortality, and life expectancy at birth. Then, we use the marginal effects of the ordered logit regression model to investigate the factors that explain club memberships.

**Results:**

The findings showed the absence of overall convergence for the three variables of interest. However, we identified the presence of convergence clubs. Moreover, we observed substantial gaps between the estimated clubs. The marginal effect results reveal that real GDP per capita, population structure, urbanization, trade, access to basic sanitation, and external health expenditure are essential to club formation. In addition, DTP immunization and the general government health expenditure as a percentage of the general government expenditure (our Abuja Declaration instrument) also play a significant role in explaining the club membership.

**Conclusion:**

The findings suggest that policymakers should develop and implement targeted club-specific health policies. Furthermore, interventions to promote increased immunization, particularly among children, should be encouraged. Governments should also make substantial efforts to increase the share of their national budget allocated to the health sector by at least 15 percent.

## Introduction

The health of people in Africa has improved substantially since 2000. The continent has witnessed a reduction in infant, under-five, and maternal mortality rates. For instance, the under-five mortality rate declined by 64 percent, from 153 in 2000 to 81 in 2015. HIV/AIDS and malaria-related mortality rates have also decreased markedly. Moreover, the region’s average life expectancy at birth is increasing, partly due to declines in adult and child mortality [1]. This progress has been reflected in African countries’ efforts toward achieving the health-related Millennium Development Goals (MDGs, hereafter) since 2000.

Several preventive, curative, and cost-effective health policy interventions have significantly improved African health outcomes. Most of these interventions were developed as part of a long-term process that predates the MDGs. However, governments and policymakers have intensively used them to meet health-related MDGs, including access to safe water and basic sanitation, skilled birth attendance, anti-retroviral therapy, and free distribution of insecticide-treated bed nets. Immunization is amongst the most cost-effective public health interventions in African countries. It has averted over a million deaths between 2000 and 2019. Since the establishment of the Expanded Programme on Immunization (EPI) in 1974 by the World Health Organization, Africa’s vaccination coverage has increased markedly. For instance, in 2019, the regional coverage of Measles was 63 percent, while the coverage of DTP3 was 66 percent. Countries with expanded coverage of the measles vaccine and DTP3 have significantly reduced child mortality [2]. Several countries have shown their commitment to increasing immunization coverage through initiatives such as Universal Childhood immunization, the Global Alliance for Vaccines and Immunization (GAVI), the Global Vaccine Action Plan (GVAP), and the WHO African Region’s EPI strategies plans of action for the periods 2001–2005 and 2006–2009 [3].

Furthermore, health spending is critical for health policy implementation. African leaders have increasingly recognized the importance of addressing health funding challenges to meet the health-related MDGs, the Sustainable Development Goals (SDGs), and, more recently, Universal Health Coverage (UHC). In this regard, the 2001 Abuja Declaration urging countries to allocate at least 15 percent of their national budgets to the health sector was a landmark, reaffirmed in the other several declarations such as the 2003 Maputo, 2009 Ouagadougou, 2012 Tunis, and the 2014 Luanda Declarations. These declarations have become prominent features in development agendas at the regional and country levels. Consequently, there has been a growing trend in public health spending in many African countries. The rapid increase in public health expenditure reflects the increased efforts at national and continental levels to ensure that relevant health interventions and plans are incorporated into preventive, curative, and treatment programmes. For instance, the increased public health spending has significantly contributed to implementing key interventions for reducing child mortality in African countries, including immunization and several programmes that improve nutrition and curb household air pollution [4].

However, Africa’s public health spending remains low compared to other regions worldwide. Several countries have shown a limited capacity to raise public funds for health, partly due to the informal nature of their economies, which makes collecting taxes difficult. In addition, most countries have not yet met the Abuja Declaration target and other health financing goals. There are considerable disparities between countries regarding allocating resources to the health sector [5].

Furthermore, Africa still bears the double burden of infectious and non-communicable diseases. The coverage of several public health interventions remains low because of weak health systems and other factors, including internal conflicts and poverty. In addition, there are substantial variations in health outcomes between countries and regions. For instance, countries in the Northern part significantly improved their populations’ health status, whereas slow progress has been recorded in the other regions. Inter-country or regional disparities in health outcomes are a significant concern among policymakers, who are faced with the double challenge of improving health status and reducing health outcome inequalities in Africa [6].

Although there is growing interest in assessing health outcome disparities, studies focusing on understanding the accurate trajectories of inter-country health outcome inequalities are rare, particularly in Africa. To our knowledge, few studies have analyzed convergence in health outcomes in Africa [7, 8] focused on convergence in health outcome indicators in the Sub-Saharan African (SSA, hereafter) region, whereas [9, 10] studied convergence in health outcomes between a group of Economic Community of West African State (ECOWAS, hereafter). However, these studies used standard convergence measures with several flaws as discussed in the literature. In contrast to analytical frameworks used in these studies, [11, 12] proposed a new method, which is adopted in the current study. A noteworthy feature of this method is that rejecting the full panel convergence null hypothesis does not imply evidence against convergence among sub-groups of countries, also known as convergence clubs.

The different rates of progress in health outcomes between African countries make it difficult to achieve SDGs 3 and 10 and the UHC. Thus, examining the progress in inter-country disparities in health outcomes will help develop better health policies to accelerate progress toward achieving common targets at the continental and global levels. Therefore, the present study assesses whether improvements in health outcomes converged in 40 African countries over the past 19 years, from 2000 to 2019. Understanding convergence in African health outcomes is critical given the region’s population size, double disease burden, mortality, and morbidity. Moreover, it will help assess the health outcomes of implementing the existing health policies and interventions. It also helps develop new policies and interventions for the health-related SDGs and UHC. Against this background, the study’s primary objective is to address the following question: Have disparities in health outcomes between African countries reduced over time? We use three health outcome indicators: infant mortality rate (IMR), the under-five mortality rate (U5M), and life expectancy at birth (LE).

The paper contributes to the literature in three ways. First, it examines the convergence hypothesis in health outcomes in Africa, which has received less attention. Second, it employs a new method proposed by Phillips and Sul (PS, hereafter) [11, 12]. It is based on a nonlinear time-varying model that incorporates the possibility of transitional divergence and allows the identification of convergence clubs. Third, the study uses the marginal effects of the ordered logit regression model to identify the factors that explain club memberships. The study incorporates control variables, including population structure, GDP per capita, urbanization, access to basic sanitation services, external health expenditure, and trade. Apart from such variables, the study investigates the role of the 2001 Abuja Declaration (measured by the general government health expenditure as a percentage of the general government expenditure), DTP immunization, and measles immunization in attaining convergence.

This paper is organized as follows: Literature and empirical review section reviews the theoretical and empirical literature. Methodology section  discusses the methodology used. Ordered logit model section  presents and discusses the empirical results. Data section provides the conclusion and policy implications.

## Literature and empirical review

The debate on economic convergence has become one of the central focuses of empirical growth literature. The notion of convergence in economics is defined as a process in which different countries become uniform over time [13], which will only happen if a catching-up occurs.

Empirical techniques for convergence tests can be classified into three main categories. The σ-convergence measures the cross-sectional dispersion. Convergence occurs when the dispersion between countries declines over time [14]. In contrast, β-convergence focuses on the cross-section correlation between the initial levels of health outcomes and their growth. There are two types of β-convergence. The absolute β-convergence implies that countries with poor health outcomes, irrespective of their economic characteristics, tend to grow faster than those with better health outcomes and thus catch up with them over time. Alternatively, the conditional β-convergence occurs when countries with different parameters converge to different steady states [15]. However, while some researchers argued that β-convergence provides a suitable testing method for convergence within a country [16], others have shown that it presents some flaws [17, 18]. For instance, [18] showed that the β-convergence collapses due to stochastic technological progress. Later, [19] revealed that the test for σ-convergence is also subject to flaws due to outliers, discontinuities, and short-run shocks. Moreover, rejecting the σ-convergence hypothesis does not necessarily mean that countries do not converge.

Lastly, stochastic convergence occurs when a country’s health outcome relative to a reference country is stationary, revealing a steady health outcome state [20]. Therefore stochastic convergence implies that differences across countries are not persistent and countries will converge in the mean zero stationary processes. However, studies have shown that in the absence of a structural break, the test of stochastic convergence may lead to misspecification errors and biased results [21].

The concept of convergence club gained popularity among health economists in recent years. The notion describes convergence among a sub-group of countries or regions with similar characteristics (e.g. density, population growth, technology) and relatively similar initial conditions [11]. Convergence club is associated with multiple equilibria.

Several empirical studies have examined the convergence in health outcomes between and within countries. The findings of these studies reveal mixed results based on the methods used. For instance, [19] tested the β-convergence hypothesis in health outcomes between 15 EU countries from 1980 to 1995. Their findings show no evidence of health outcome convergence. Using the methodology proposed by [11, 22] also found no convergence in health outcomes between 19 OECD countries from 1972 to 2006. In contrast, employing the β-convergence and Johansen Co-integration technique, [9] found that health outcomes converged for members of the Economic Community of West African State (ECOWAS) between 1995 and 2011. Similarly, using the β- and σ-convergence tests, [7] found evidence of σ-convergence in under-five mortality rate over 1990–2002 for 46 SSA. The selected countries’ life expectancy at birth converged between 1990 and 2011 when considering the differences in country-specific factors.

Furthermore, [10] employed the β- and σ-convergence methods to investigate the convergence hypothesis in income per capita and health outcomes. The findings indicate the presence of β-convergence for all the variables of interest, while there was no evidence of σ − convergence in life expectancy at birth. Using the Generalized Method of Moments and the Pooled Least Squares, they found that trade and governance significantly affect convergence in health outcomes. Similarly, [23] also found evidence of β − and σ − convergence in life expectancies between the EU members and the Spanish provinces during the 1998–2018 period. On the other hand, [24] found evidence of absolute β-convergence in life expectancy between the main Indian States from 1981 to 2015. However, the β- and σ-convergence in child and maternal mortality were only evident after 2000. Their results also revealed the absence of convergence in child underweight.

In sum, the existing literature showed that studies on health outcomes convergence mainly focused on OECD and EU countries. Very few studies have been done in Africa. Moreover, no African health outcome convergence studies account for transitional dynamics or paths to our knowledge. Also, previous studies do not consider the possible existence of convergence clubs. Therefore, this study contributes to the literature on health outcome convergence in Africa using the methodology proposed by [11, 12]. This method is suitable for this study because it considers the possibility of individual and transitional heterogeneity or divergence. It is a nonlinear time-varying model which does not rely on trend stationarity or stochastic non-stationarity assumptions.

Moreover, the method includes a simple “log t” test with the clustering and club merging procedures, which may have several policy implications. The study also contributes to the literature on the field by using the marginal effects of the ordered logit model to identify the factors that explain club memberships. In doing so, we will examine the roles of immunizations and the 2001 Abuja Declaration policy to allocate at least 15 percent of government budgets to health.

## Methodology

The theoretical framework of this study is derived from the neoclassical growth theory. The convergence hypothesis in health outcomes is tested following the $\mathrm{log}t-test$, the clustering algorithm, and the club merging procedures developed by [11, 12]. The methodology adopts the following time-varying common-factor representation for ${Q}_{it}$ country i.

1 $$Q_{it}=x_{it}\mu_t,\;for\;all\;i,t$$

where ${\mu }_{t}$ represents the common component and ${x}_{it}$ is a time-varying systematic idiosyncratic component that varies over time and across the cross-section. The idiosyncratic element measures the distance between ${Q}_{it}$ and the common component ${\mu }_{t}$. Moreover, in this dynamic formula,${x}_{it}$ becomes the transition path to the common steady-state growth path determined by ${\mu }_{t}$. The estimation of ${x}_{it}$ provides information about the transition behavior of particular panel units [11]. showed that the estimation of the parameter ${x}_{it}$, as well as additional structural restrictions and assumptions must be imposed. Thus, they propose the construction of the following relative transition parameter as a viable way to model ${x}_{it}$.

2 $${b}_{it}= \frac{{Q}_{it}}{\frac{1}{N}\sum_{i=1}^{N}{Q}_{it}}= \frac{{ x}_{it}}{\frac{1}{N}\sum_{i=1}^{N}{ x}_{it}}$$

where ${b}_{it}$ is the transaction path because it traces out an individual trajectory for each country $i$ relative to the panel average. It also measures the relative departure of country $i$ from the common steady-state growth path ${\mu }_{t}$. As a result, the transition path ${b}_{it}$ shows the divergence from ${\mu }_{t}$. Whenever, the panel units converge, the relative transaction path ${b}_{it}$ converges to unity: ${b}_{it} \to 1$ for all $i=1,\dots ,N$ as $t\to \infty$. The cross-sectional variation ${B}_{it}$ of the transition path converges to zero, as shown below:

3 $$B_t=\frac1N\sum_{i=1}^N(h_{it}-{1)}^2\rightarrow0,\;as\;t\rightarrow\infty$$

By construction, the mean of the relative transition path ${b}_{it}$ is unity. Phillips et. al [11] proposes a semi-parametric model for ${x}_{it}$ to order to construct a formal statistical test for convergence

4 $${x}_{it}= {x}_{i}+ \frac{{\tau }_{i} {\varphi }_{it}}{{L\left(t\right)t}^{a}}$$

where ${x}_{it}$ is fixed, ${\varphi }_{it}$ are $iid$ N(0, 1) across $i$, ${\tau }_{i}$ are idiosyncratic scale parameters, $L\left(t\right)$ is a slowly varying increasing function $\mathrm{log}(t)$, so that $L\left(t\right)$ ⇾∞ as $t$ ⇾∞.The parameter $a$ represents the speed of convergence, i.e. the rate at which the cross-sectional variation decays to zero. As a result, ${xi}_{it}$ converges to ${x}_{i}$ for all $a \ge 0$. In the framework, the null hypothesis is ${H}_{0}: { x}_{i}$ = $x$ and $a \ge 0$, while the alternative is ${H}_{1}: { x}_{i}$ ≠ $x$ for all i, or $a<0$. The null hypothesis implies convergence for all countries, while the alternative hypothesis implies no convergence for some countries. In addition, the alternative hypothesis also accommodates the overall divergence and the club convergence. To test the presence of convergence for a sample of countries, PS use the following log $t$ specification:

5 $$\mathrm{log}\left(\frac{{B}_{1}}{{B}_{t}}\right)-2logL\left(t\right)=\widehat{e}+ \widehat{x}logt+{\mu }_{t}, t=\left[rT\right],\left[rT\right]+1\dots ,T$$

where ${B}_{t}$ is the cross-sectional variation. $\frac{{B}_{1}}{{B}_{t}}$ represents the ratio of the cross-sectional variation at the beginning of the sample ${B}_{1}$ (i.e. ${B}_{t}$ at t = 1) divided by the respective variation for every point in time t, suggesting ${B}_{t}$ (t,…,T). $L\left(t\right)$ is $\mathrm{log}(t)$, and -$2log\left(log t\right)$ is the penalization function that improves the performance of the test under the alternative. $r>0$, and it assumes an interval [0,1] to discard the first block of observation from the estimation, and [rT] is the integer part of rT. Thus, PS proposes setting $r$ є [0.2, 0.3] for T < 50 because the extensive Monte Carlo simulations indicate that this choice of $r$ is stationary in terms of the size and power properties of the test. The null hypothesis of convergence is tested using a one-sided t-test. This test is robust to heteroscedasticity and autocorrelation of the inequality $a\ge 0$ (using the estimated $\widehat{x}= 2a$). The convergence of the full sample requires that $\widehat{x}$ is either positive or equal to zero. When the conventional t-statistics ${t}_{x}$ is used, the null hypothesis of convergence is supported if ${t}_{x}> -1.65$. PS (2007) proposes to use the clustering procedure if the null hypothesis is rejected to identify possible subgroups of countries. The process is summarized as follows.

1. Ordering countries according to the last panel observation of the period.
2. Identifying a base group of $g$ countries with the highest health outcomes in the panel to form the subgroup ${W}_{k}$ for some $N>g\ge 2$. Then, run the $log t$ regression, and the convergence test statistic ${t}_{x}(g)$ is calculated for all the different values of $g$. The core group size ${g}^{*}$ is selected by maximizing ${t}_{x}$ over $g$ following the minimum $\left\{{t}_{\widehat{b}}(k)\right\}\mathrm{criteria}>-1.65$. If minimum ${t}_{C}>-1.65$ holds for all sequential pairs, then a core convergence sub-group exists.
3. Adding each of the remaining countries separately to the *b*ase group and employing the ${Log t}_{x}$ for each additional country. The additional country satisfies the membership condition if ${t}_{x}> -1.65$, then the country is included in the core group.
4. Repeating steps 1–3 on the remaining countries to determine whether the group can be subdivided into convergence clusters [12] pointed out that using the sign criterion in the second step of the clustering procedure may lead to overestimating of the number of initial convergence clubs. Therefore, they proposes performing a club merging test after the clustering algorithm. If ${t}_{x}$> -1.65, the initial clubs are merged at the five percent significance level, forming a larger club.

### Ordered logit model

Though the clustering approach proposed by [11, 12] can be used to identify convergence clubs, it does not explain how such clubs are formed. Consequently, the current study also attempts to identify the underlying factors that drive club formation and quantify the effects of these factors across the clubs.

Following the procedure of PS, the final clubs obtained are ordinal variables ordered meaningfully [11, 12]. Hence, we use the ordered logit model to investigate the determinants of club membership for the selected African countries. This method provides a means to assess the ordering information compared to the multinomial logistic regression which tends to ignore the ordered aspect of the clubs. The ordered logit model has been employed in various fields [25].

We consider ${Y}_{i}$ the ordinal response variable and $G$ the countries ${i}^{^{\prime}}s$ convergence clusters. Let ${Q}_{i}$ denotes the explanatory variable vectors that affect club formation and ${P}_{ig}$ represents the probability that a country $i$ is a member of the dependent variable final club $g$. The cumulative probabilities are expressed in Eq. (6) if the final clubs are ordered in the sequence $g=1,\dots , G.$

6 $${H}_{ig}=K({Y}_{i}\le \frac{{Y}_{g}}{{Q}_{i}})$$

The ordered logit regression model provides a link between the explanatory variables and the probability of the final clubs $G$ described by a set of $G-1$ equations based on the cumulative probabilities as follows:

7 $$\mathrm{log}\left(\frac{{H}_{ig}}{1-{H}_{ig}}\right)={\epsilon }_{g}- \alpha {Q}_{i }, g=1, 2, \dots \dots , G-1$$

where ${\alpha }_{1}{Q}_{i1}+ {\alpha }_{2}{Q}_{i2}+\dots {+ \alpha }_{n}{Q}_{in}$ represent the total number of explanatory variables. The likelihood of belonging to a given club is determined using ${Q}_{i}$’s mean value and is presented as follows:

8 $$p\left(\gamma=\frac1{Q_i}\right)=\frac{\exp\left(\epsilon_g-\alpha Q_i\right)}{1+\exp\left(\epsilon_g-\alpha Q_i\right)},\;for\;g=1$$

9 $$p\left(\gamma=\frac g{Q_i}\right)=\frac{\exp\left(\epsilon_g-\alpha Q_i\right)}{1+\exp\left(\epsilon_g-\alpha Q_i\right)}-\frac{\exp\left(\epsilon_{g-1}-\alpha Q_i\right)}{1+\exp\left(\epsilon_{g-1}-\alpha Q_i\right)},\;for\;c=2,\dots\dots,G-1$$

10 $$p\left(\gamma=\frac G{Q_i}\right)=1-\frac{\exp\left(\epsilon_{g-1}-\alpha Q_i\right)}{1+\exp\left(\epsilon_{g-1}-\alpha Q_i\right)},\;for\;G$$

The current study analyses the marginal effects of the predicted probabilities to identify the factors that predict club formation. The marginal effects show the probability of belonging to a final club when an independent variable changes by one unit, while all the other variable are held constant. They also provide meaningful information about the relationship between the dependent and independent variables. Previous studies have shown that the ordered logit regression results do not give a clear insight into the magnitude of the link between the variables [25]. We will provide results from the multinomial logistic regression for the robustness check.

### Data

The study uses annual panel data for 40 African countries extracted from the World Development Indicators (WDI). The period of study spans from 2000 to 2019. We did not have issues related to missing observations. We constructed the external resources for health as a percentage of GDP as follows: First, we multiplied the external health expenditure per capita (PPP) by the total population. Secondly, we divided the results with the GDP (PPP), which was also extracted from the WDI. Appendix 1, Panel A and B provide information about the data and the list of countries, respectively.

## Estimation results and discussion

### Descriptive statistics

Table 1 reports the descriptive statistics of all the variables used. The results display a high level of consistency because the mean values for all variables fall within the minimum and maximum values. On average, under-five and infant mortality rates were 90 and 58 deaths per 1,000 live births, respectively, which is relatively high and far from the Sustainable Development target of 25 deaths per 1,000 live births [26]. The average life expectancy at birth was approximately 59 years, with a maximum of 77 years. On average, real GDP per capita was US$5316.77, ranging from US$715.45 to US$41249.49. The standard deviation of US$5767.77 shows significant disparities in income distribution in the continent. Approximately 42 percent of the African population lives in urban areas, with a maximum of 90 percent showing Africa’s rapid urbanization. On average, trade represents 66 percent of Africa’s GDP, ranging from 1.22 to 175.80 percent. The average number of individuals using at least basic sanitation services is approximately 37 percent of the total population, which remains low compared to other regions worldwide. Roughly 3 percent of the African population is 65 years and above, while approximately 41 percent is below 15 years. These figures align with the findings of [27], which revealed that Africa has the fastest-growing youth population in the world. On average, approximately 74.16 percent of children ages 12–23 months were immunized against measles, while 76.55 percent were immunized against DPT. However, Africa still lags behind the global immunization coverage targets of at least 90 percent DTP coverage nationally and 80 percent DPT coverage in every district [3]. The average of the general government health expenditure as a percentage of general government expenditure (the Abuja declaration policy instrument) was approximately 7. 09 percent, below the target of 15 percent envisaged in the Abuja policy. This figure shows that several African countries have not yet implemented the policy.

**Table 1:** Descriptive statistics of variables of the full sample

Variables	Mean	Std. Dev	Min	Max
**Dependent variables**				
Under-5 mortality rate (U5M)	90.421	43.835	14.500	224.900
Infant mortality rate (IMR)	58.376	24.767	12.500	138.100
Life expectancy at birth (LE)	58.852	7.709	39.441	76.880
**Independent variables**				
Real GDP per capita (RGDp)	5316.538	5767.766	715.454	41249.490
Population above 65 year old (POPG65)	3.378	1.398	1.871	11.999
Population below 15 years old (POPL15)	41.267	6.488	17.260	50.264
Urban population (URB) (UBPOP)	42.218	16.900	8.246	89.741
External health expenditure (%of GDP) (EXHE)	.208508	0.016	0	0.537
Trade (TO)	66.361	28.426	1.219	175.798
People using at least basic sanitation service (BASS)	36.508	23.792	4.192	96.377
Measles immunization (IMEAS)	74.164	17.972	16.000	99.000
DPT immunization (IDTP)	76.545	18.636	19.000	99.000
Domestic general government health exp. (DGGHE)	7.079	3.490	0.633	18.287

Table 1 provides the descriptive statistics of the variables used in the study. The first three rows provide the descriptive statistics of the dependent variables, while the remaining rows provide the descriptive statistics of the independent variables. It is important to note that the explanatory variables are used to assess the factors that explain club formation. Source: Authors’ computation from WDI data (World Bank)

### Convergence analysis

Panels A, B, and C of Table 2 report the log-t and club clustering results for all the health outcomes variables used in the study. The first rows report the results for full sample convergence, while the other rows display the results of the club clustering. The t-statistics obtained through the log t-test of IMR, U5M, and LE are -264.13, -255.30, and -3.17, respectively, less than the critical value (-1.65). Therefore, the null convergence hypothesis for the selected sample is rejected at the 1 percent significance level for all the health outcome variables. These results suggest that inter-country disparities in health outcomes widened over time. The findings are consistent with [22], who found no evidence of convergence in life expectancy between OECD countries. However, [7] showed the presence of the σ-convergence in under-five mortality rate and life expectancy at birth between 46 SSA from 1990 to 2011.

**Table 2:** Convergence and final club classification results

Sample	Countries	B^ *Coeff*	SE	t-stat
**Panel A: Infant mortality rate log-t and club clustering test results**				
Overall (40)	All the countries in the sample	-1.025^a^	0.004	-264.129
Cub 1 (3)	Central African Republic | Nigeria | Sierra Leone	0.530	0.130	4.069
Club 2 (3)	Benin | Congo, Dem. Rep. | Guinea	0.161	0.072	2.230
Club 3 (5)	Botswana | Cote d'Ivoire | Equatorial Guinea | Mali | Mauritania	0.055	0.060	0.909
Club 4 (6)	Angola | Burkina Faso | Cameroon | Comoros | Guinea-Bissau | Togo	0.967	0.071	13.586
Club 5 (8)	Burundi | Gambia, The | Madagascar | Namibia | Niger | Sudan | Eswatini | Zambia	0.214	0.048	4.488
Club 6 (5)	Gabon | Ghana | Kenya | Tanzania | Uganda	1.078	0.089	12.109
Club 7 (5)	Algeria | Mauritius | Rwanda | Senegal | South Africa	0.177	0.077	2.302
Club 8 (3)	Cabo Verde | Morocco | Tunisia	1.001	0.167	6.000
Non-convergent gr. (2)	Chad | Congo, Rep	-1.624^a^	0.025	-64.995
**Panel A1: Club Merging results**				
Club 1 + 2		-0.610^a^	0.032	-19.311
Club 2 + 3		-0.089	0.048	-1.838
Club 3 + 4		0.134	0.046	2.920
Club 4 + 5		-0.342^a^	0.022	-15.493
Club 5 + 6		-0.191^a^	0.016	-11.973
Club 6 + 7		-0.164^a^	0.052	-3.175
Club 7 + 8		-0.200^a^	0.039	-5.199
**Panel A2: Final club classifications results**				
Final club 1 (3)	Central African Republic | Nigeria | Sierra Leone	0.530	0.130	4.069
Final club 2 (3)	Benin | Congo, Dem. Rep. | Guinea	0.161	0.072	2.230
Final club 3 (11)	Angola | Botswana | Burkina Faso | Cameroon | Comoros | Cote d'Ivoire | Equatorial Guinea | Guinea-Bissau | Mali | Mauritania | Togo	0.134	0.046	2.920
Final club 4 (8)	Burundi | Gambia, The | Madagascar | Namibia | Niger | Sudan | Eswatini | Zambia	0.214	0.048	4.488
Final club 5 (5)	Gabon | Ghana | Kenya | Tanzania | Uganda	1.078	0.089	12.109
Final club 6 (5)	Algeria | Mauritius | Rwanda | Senegal | South Africa	0.177	0.077	2.302
Final club 7 (5)	Cabo Verde | Morocco | Tunisia	1.001	0.167	6.000
Non-convergent gr. (2)	Chad | Congo, Rep	-1.624^a^	0.025	-64.995
**Panel B: Under-5 mortality rate log-t and club clustering test results**				
Overall (40)	All the countries in the sample	-1.004^a^	0.004	-255.300
Cub 1 (5)	Central African Republic | Chad | Guinea | Nigeria | Sierra Leone	0.2224	0.082	2.711
Club 2 (7)	Benin | Burkina Faso | Congo, Dem. Rep. | Cote d'Ivoire | Equatorial Guinea | Mali | Mauritania	0.008	0.015	0.545
Club 3 (7)	Angola | Cameroon | Comoros | Guinea-Bissau | Niger | Sudan | Togo	0.642	0.082	7.826
Club 4 (9)	Botswana | Burundi | Gambia, The | Ghana | Madagascar | Namibia | Eswatini | Tanzania | Zambia	0.348	0.086	4.036
Club 5 (6)	Congo, Rep. | Gabon | Kenya | Rwanda | Senegal | Uganda	0.393	0.220	1.791
Club 6 (6)	Algeria | Cabo Verde | Mauritius | Morocco | South Africa | Tunisia	0.164	0.079	2.071
**Panel B1: Club Merging results**				
Club 1 + 2		-0.935^a^	0.014	-65.508
Club 2 + 3		-0.221^a^	0.021	-10.553
Club 3 + 4		-0.480^a^	0.026	-18.484
Club 4 + 5		-0.323^a^	0.050	-6.491
Club 5 + 6		-0.413^a^	0.028	-14.973
**Panel B2: Final club classifications**				
Final club 1 (5)	Central African Republic | Chad | Guinea | Nigeria | Sierra Leone	0.2224	0.082	2.711
Final club 2 (7)	Benin | Burkina Faso | Congo, Dem. Rep. | Cote d'Ivoire | Equatorial Guinea | Mali | Mauritania	0.008	0.015	0.545
Final club 3 (7)	Angola | Cameroon | Comoros | Guinea-Bissau | Niger | Sudan | Togo	0.642	0.082	7.826
Final club 4 (9)	Botswana | Burundi | Gambia, The | Ghana | Madagascar | Namibia | Eswatini | Tanzania | Zambia	0.348	0.086	4.036
Final club 5 (6)	Congo, Rep. | Gabon | Kenya | Rwanda | Senegal | Uganda	0.393	0.220	1.791
Final club 6 (6)	Algeria | Cabo Verde | Mauritius | Morocco | South Africa | Tunisia	0.164	0.079	2.071
**Panel C: Life expectancy at birth log-t and club clustering test results**				
Overall (40)	All the countries in the sample	-0.108^a^	0.034	-3.165
Cub 1 (19)	Algeria | Angola | Botswana | Burundi | Congo, Rep. | Gabon | Kenya | Morocco | Namibia | Niger | Rwanda | Senegal | Sierra Leone | South Africa | Eswatini | Tanzania | Tunisia | Uganda | Zambia	0.321	0.068	4.690
Club 2 (8)	Burkina Faso | Cabo Verde | Congo, Dem. Rep. | Guinea | Madagascar | Mali | Mauritius | Sudan	0.098	0.048	2.049
Club 3 (13)	Benin | Cameroon | Central African Republic | Chad | Comoros | Cote d'Ivoire | Equatorial Guinea | Gambia, The | Ghana | Guinea-Bissau | Mauritania | Nigeria | Togo	-0.050	0.054	-0.913
**Panel C1: Club Merging results**				
Club 1 + 2		0.293	0.061	4.852
Club 2 + 3		-0.153^a^	0.043	-3.557
**Panel C2: Final club classifications**				
Final club 1 (27)	Algeria | Angola | Botswana | Burundi | Congo, Rep. | Gabon | Kenya | Morocco | Namibia | Niger | Rwanda | Senegal | Sierra Leone | South Africa | Eswatini | Tanzania | Tunisia | Uganda | Zambia | Burkina Faso | Cabo Verde | Congo, Dem. Rep. | Guinea | Madagascar | Mali | Mauritius | Sudan	0.293	0.061	4.852
Final club 2 (13)	Benin | Cameroon | Central African Republic | Chad | Comoros | Cote d'Ivoire | Equatorial Guinea | Gambia, The | Ghana | Guinea-Bissau | Mauritania | Nigeria | Togo	-0.050	0.054	-0.913

^a^indicates rejection of the null hypothesis of convergence and club convergence merging. *SE* Standard error. Panels A, B, and C present the convergence of results of infant, under-five mortality rates, and life expectancy at birth, respectively. The first rows provide the results of the full sample, while the other rows provide the results of the convergence clubs. Then, the results from the club merging results and final club classification are provided for each variable

^signifies the rejection of the null hypothesis of convergence, club merging, and final club classification

Source: Authors’ computation using WDI data

The PS club clustering test on the full panel reveal eight, six, and three initials clubs for IMR, U5M, and LE, respectively. Moreover, we find two diverging countries for IMR: Chad and Congo Republic. These countries are not members of any club. However, the club merging algorithm was performed on the initials clubs found to avoid overestimating the clubs. The club merging results found in Panel A1, B1, and C1 of Table 1 does not lead to any amalgamation of the initial clubs for the U5M model. In contrast, the initial number of clubs for the IMR and LE models is reduced to seven and two, respectively. The final club classifications are reported in Panel A2, B2, and C2 of Table 2, with seven, six, and two final clubs for the IMR, U5M, and LEB models, respectively. Panopoulou E et. al [22] also identified convergence clubs in OECD countries’ life expectancies and crude mortality rates. The $\widehat{x}$ values for all the final clubs (except final club 2 for the LE model) indicate that final club members converge conditionally. However, the final club 2 of the LE model is a weak convergence club, given the negative value of the estimated coefficient [12].

The mean values of the final clubs reported in Table 3 indicate the presence of disparities between the final clubs. The differences are well pronounced for the infant and under-five mortality rates. For instance, the results show that countries in final club 7 performed relatively well, with an average IMR estimated at 22 deaths per 1,000 live births, which is below the SDG target of 25 deaths per 1,000 live births [27]. However, countries in all the other clubs, including the divergent group, are still far from meeting the global target of 25 death per 1,000. Final club 1 had the worst IMR, accounting for 99 deaths per 1,000 live birth, ranging from 74.00 to 138.10 deaths per 1,000 live births.

**Table 3:** Averages of health outcome measures by final clubs, 2000–2019

	**Infant mortality rate**			**Under-5 mortality rate**			**Life expectancy at birth**		
**Clubs**	**Mean** **(Sd. Dev.)**	**Min**	**Max**	**Mean** **(Sd. Dev.)**	**Min**	**Max**	**Mean** **(Sd. Dev.)**	**Min**	**Max**
Final club 1	98.528 (15.899)	74.000	138.100	145.100 (27.788)	98.000	224.900	60.259 (8.356)	39.441	76.880
Final club 2	77.843 (12.253)	57.900	106.500	116.506 (25.583)	73.000	187.400	55.931 (5.033)	44.061	64.925
Final club 3	68.946 (17.470)	36.900	121.500	107.782 (36.356)	58.400	224.900			
Final club 4	53.853 (14.722)	30.900	97.000	77.436 (25.552)	41.900	155.900			
Final club 5	47.800 (12.370)	31.500	87.100	73.954 (29.085)	39.700	185.200			
Final club 6	35.420 (19.347)	12.500	109.500	30.112 (16.099)	14.500	79.200			
Final club 7	22.428 (7.678)	12.800	43.900						
Divergent gr	66.665 (21.414)	34.700	99.700						
**Total**	**58.376** **(24.767)**	**12.500**	**138.100**	**90.421** **(43.835)**	**14.500**	**224.900**	**58.852** **(7.709)**	**39.441**	**76.880**

Table 3 presents average values of the dependent variables by final club. The standard deviations are in parenthesis. The Table also present the minimum and maximum for each club. Source: Author’s own calculation

**Fig. 1:**
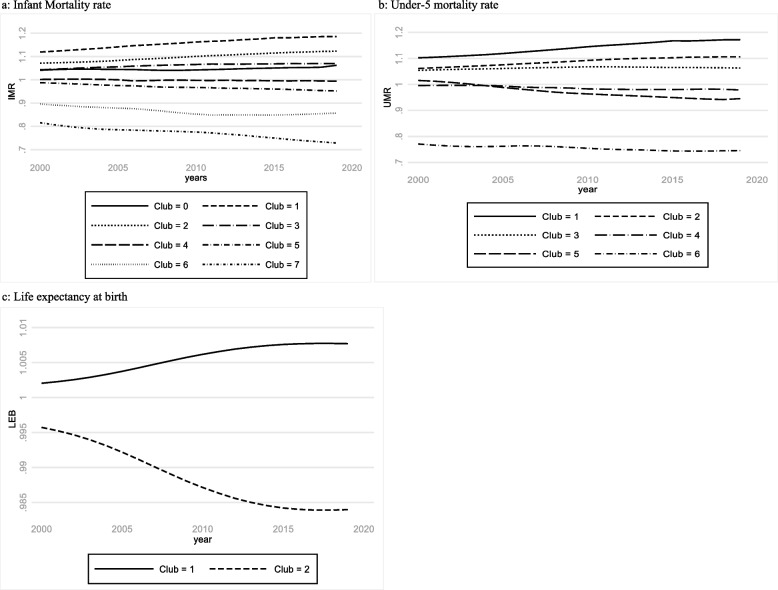
Averages of health outcomes by final clubs, 2000–2019. Figure 1 shows the average values of the dependent variables by final clubs. **a** shows the average values for infant mortality rate. **b** shows the average values for under-five mortality rate. Figure 1c show the average values for life expectancy at birth

Furthermore, the U5M model results also show significant between-club differences. No club has met the global target of 25 deaths per 1,000 live births. However, final club 6 seems to perform relatively better compared to the rest of the final clubs. The worst outcomes are recorded in final clubs 1, 2, and 3, with U5M above 105 deaths per 1,000 live births, indicating that countries in these final clubs have made little effort to reduce U5M between 2000 and 2019. The variation between the final clubs for the LE model is relatively small, with a gap of approximately four years. The average LE for countries in final club 1 is about 60 years, while that for final club 2 is about 56 years. Figure 1, which displays the differences between the average transitional behaviors of the final clubs for each variable of interest, supports the findings regarding the mean values of the final clubs presented in Table 3.

## Determinants of club membership results

The PS method allows us to identify the presence of convergence clubs. Still it does not provide insight into the country-specific characteristics that could affect their probabilities of belonging to a convergence club. Consequently, we use the ordered logit to assess the determinants of club formation. The statistical analysis of the three models reported in Table 4 shows that the Wald Chi2 values are significant at a 5 percent level (Prob.Chi2 = 0.0000), indicating that the models are of good fit and they perform well. The estimated coefficients in the ordered logit models are in log-odds form. A positive odds ratio implies an increase in the likelihood of convergence into a particular final club. In contrast, a negative odds ratio reduces the probability of belonging to final clubs, given a unit increase in the variables of interest.

**Table 4:** Ordered logit Results

Variables	(1) Infant mortality rate model	(2) Under-five mortality rate model	(3) Life expectancy at birth model
	Coefficients (Std. Err.)	Coefficients (Std. Err.)	Coefficients (Std. Err.)
Log real GDP per capita	0.135 (0.153)	-0.285^b^ (0.131)	-0.422^b^ (0.204)
Log population below 15 year old	-2.043 (1.547)	-11.556^a^ (1.620)	-4.151^b^ (1.609)
Log population above 65 year old	1.418^c^ (0.737)	-1.886^b^ (0.771)	-3.133^a^ (0.706)
Log urban population	-0.481^b^ (0.212)	-0.182 (0.185)	2.667^a^ (0.377)
Log external health expenditure	-3.555^b^ (1.615)	-2.877 (1.930)	1.581 (1.599)
Log trade	0.085 (0.165)	0.414^a^ (0.097)	-0.912^a^ (0.231)
Log people using at least basic sanitation service	1.172^a^ (0.159)	0.783^a^ (0.144)	-0.886^a^ (0.214)
Log domestic general government health expenditure	1.324^a^ (0.159)	1.044^a^ (0.163)	-1.060^a^ (0.215)
Log measles immunization	1.143 (0.801)	0.212 (0.785)	-0.279 (0.870)
Log DPT immunization	2.395^b^ (0.755)	2.753^a^ (0.833)	-1.352^c^ (0.813)
Number of obs	787	787	787
Wald chi2(10)	643.770	623.960	175.25
Prob > chi2	0.0000	0.0000	0.0000
Pseudo R2	0.244	0.276	0.232
Log pseudolikelihood	-1153.0021	-1011.3771	-378.989

^a^, ^b^, and ^c^ represent statistical significance at 1%, 5%, and 10%, respectively. The standard errors are in parentheses. The first column presents the independent variables considered. The Second column presents the ordered logit results for the infant mortality rate model, the third column provides the under-five mortality rate model results, and the fourth column provides the results for the life expectancy model. Source: Authors’ computations

To interpret the impacts of the variables, Table 5 in Appendix 2 reports the marginal effects computed at the mean value of the variables. Marginal effects provide meaningful information about the impacts of each variable on the change in the likelihood of belonging to a club given a unit change in the variable.

### Infant mortality rate marginal probability results

The results of the initial population above 65 years old indicate that a unit increase in the variable decreases the probability of converging into final clubs 1, 2, 3, and the divergent group by 0.06, 0.04, 0.05, and 0.05 log points, respectively. Yet, it increases the likelihood of converging into final clubs 4, 5, 6, and 7 by 0.03, 0.04, 0.06, and 0.07 log points, respectively. Similarly, an increase in initial population using at least basic sanitation services reduces the likelihood of membership to final clubs 1, 2, 3, and the diverging group. Still, it increases a country’s probability of belonging to final clubs 4, 5, 6, and 7. In contrast, the estimated results for urban population and external health expenditure reveal that a unit increase in the variables increases the probability of affiliation to final clubs 1, 2, 3, and the divergent group but reduces the likelihood of belonging to final clubs 4, 5, 6, and 7. These results suggest that countries with increased urban populations and external health expenditure are likelier to belong to the final clubs with the worst average infant mortality rates. This might suggest that efforts toward increasing external health expenditure in African countries with high rates of infant mortality are yielding the expected results. This might be explained by high corruption and mismanagement of funding, particularly in the health sector [28, 29]. The results for the other control variables are insignificant.

The marginal effect results of the variables of interests reveal that a unit increase in initial log IDPT is associated with a lower likelihood of belonging to final clubs 1, 2, 3, and the diverging group (0.09, 0.07, 0.08, and 0.09 log points, respectively) and a higher probability of belonging to final clubs 4, 5, 6 and 7 by 0.05, 0.07, 0.11, and 0.11 log points, respectively. In 2019, most countries in final clubs 1, 2, and 3 had less than 80 percent of DTP3 coverage with many unvaccinated infants. Nigeria alone had about 3 million unvaccinated infants. However, most countries in final clubs 6 and 7 are characterized by 90 percent or above of DPT3 vaccination coverage. For instance, Cabo Verde, Morocco, Tunisia, and Ghana had over 90 percent of DTP3 vaccination coverage and few unvaccinated infants [2, 30]. Furthermore, a unit increase in initial domestic general government health expenditure as a percentage of general government expenditure (the Abuja instrument) is associated with a lower likelihood of membership to final clubs 1, 2, 3, and the diverging countries by 0.05, 0.04, 0.05, and 0.05, log point, respectively. But it increases the likelihood of affiliation to final clubs 4, 5, 6, and 7 by 0.03, 0.04, 0.06, and 0.06 log points, respectively. These results suggest that countries with increased initial public health spending and DPT immunization coverage will likely belong to final clubs with lower infant mortality rates. The results for measles immunization are insignificant.

### Under-five mortality rate marginal effect results

Under-five mortality rate model’s marginal effect results suggest a higher likelihood of affiliation with final clubs 1, 2, and 3 of 0.02, 0.01, and 0.03 log points, respectively, for a unit increase in initial real GDP per capita. But a lower probability of belonging to final clubs 4, 5, and 6 by 0.01, 0.01, and 0.02 log points, respectively. Similarly, an increase in the initial population below 15 years old by one unit is associated with a higher probability of membership to the final clubs 1, 2, and 3 by 0.93, 0.58, and 0.11 log points, respectively. Still, it reduces the likelihood of converging into final clubs 4, 5, and 6 by 0.43, 0.55, and 0.65 log points, respectively. A similar pattern is observed when the population above 65 years old increases by a unit. However, a unit increase in initial trade and the population using at least basic sanitation services leads to a lower likelihood of membership to final clubs 1, 2, and 3 but a higher probability of belonging to final clubs 4, 5, and 6. The rest of the control variables are insignificant.

When analyzing the variables of interest, the results reveal that an increase in initial DTP immunization among children of 12–23 months lowers the probability of membership to final clubs 1, 2, and 3 by 0.22, 14, and 3 log points, respectively. But it raises the likelihood of affiliation to final clubs 4, 5, and 6 by 0.10, 0.13, and 0.16 log points, respectively. Similarly, an increase in initial log domestic general government health expenditure as a percentage of general government expenditure reduces the likelihood of belonging to final clubs 1, 2, and 3 by 0.08, 0.05, and 0.01 log points, respectively. However, it increases the probability of membership to final clubs 4, 5, and 6 by 0.0.04, 0.05, and 0.06, respectively. The results for measles immunization remain insignificant.

### Life expectancy marginal effect results

The results show that a unit increase in initial real GDP per capita increases the probability of membership to final club 1 by 0.07 log points. But it reduces the likelihood of affiliation to final club 2 by 0.07 log points. Similarly, there is a higher probability of converging into final club 1 and a lower one of belonging to final club 2 when variables such as population below 15 years old, population above 65 years old, trade, and population using at least basic sanitation services increase by one unit. However, an increase in urban population decreases the probability of belonging to final club 1, but raises the likelihood of membership in final club 2. The other control variables show insignificant results.

The marginal effects of measles immunization are insignificant. However, a unit increase in initial DTP immunization among infants aged 12 to 23 months is associated with a higher probability of membership to final club 1 (0.22 log points) but a lower likelihood of affiliation to final club 2 of 0.22 log points. Similarly, as initial domestic general government health expenditure as a percentage of general government expenditure increases by one unit, there is a higher probability of belonging to final club 1 (0.17 log points) and a lower likelihood of membership to final club 2 of 0.17 log points.

### Discussion of the empirical results

Our findings do not support the hypothesis of full sample convergence in infant mortality rate, under-five mortality rate, and life expectancy at birth for the 40 selected African countries from 2000 to 2019. Therefore, inter-country disparities in health outcomes remain persistent and pervasive in Africa. We identify the presence of convergence clubs for each variable. However, significant gaps exist between the final clubs. The gaps are well pronounced for infant and under-five mortality rates. In both cases, the members of final clubs 1, 2, and 3 had the highest mortality rates among children. This suggests that these countries have not made substantial efforts to meet the global sustainable target of 25 deaths per 1,000 live births. According to [6], weak political will, internal conflicts, limited public funding, and political instability are the main drivers of high mortality and morbidity in Africa, particularly among children. In addition, the persistent inter-country disparities in health outcomes have high financial costs, and they tend to slow development In Africa. In European Union, the losses linked to health outcome disparities is estimated at 1.4 percent of the GDP, which is low compared to the African region [31].

The marginal effect results suggest that a unit increase in the population using basic sanitation services and people above 65 years is associated with a high probability of belonging to the final clubs with a lower average infant mortality rate. Trade and access to basic sanitation services increase the likelihood of membership to final clubs with lower under-five mortality rates and life expectancy at birth. In contrast, increased urbanization and external health expenditure positively impact the probability of converging to the final clubs with the worst average health outcomes. The rapid and unplanned urbanization in most African countries (particularly those belonging to the final clubs with the worst health outcomes) is fraught with health hazards, including air pollution, insufficient drinking water, poor access to basic sanitation, stress resulting from high unemployment, and poverty, among others [6]. These findings broadly confirm the results of studies by [8] and [10], who found that trade, income, and population structure lead to convergence in health outcomes.

Moreover, for all three models, DTP immunization and public health spending are also important drivers of club membership. The overall results suggest that increases in these variables positively affect the likelihood of joining final clubs with better health outcomes. However, it decreases the probability of belonging to clubs with worse health outcomes. These findings align with the report by [5] that immunization is a cost-effective public health intervention that substantially reduces mortality and morbidity among children. Moreover, countries with low immunization coverage have the highest mortality rate among children. For instance, the coverage of DTP immunization in Nigeria, The Central African Republic, Chad, and the Republic of Congo was below 60 percent in 2019. Several factors, including population size, political instability, and internal conflict, restrict health services activities, leading to low vaccination and immunization coverage [32]. However, Zambia, Burundi, Eswatini, and Namibia (all belonging to final clubs with relatively better health outcomes) had a DTP immunization coverage of over 90 percent [33].

Furthermore, the 2001 Abuja Declaration policy is essential to club formation. According to [5], nineteen countries have reduced their relative government allocation to health since 2000. These include Benin, Sao Tome and Principe, Botswana, Cameroon, Comoros, Equatorial Guinea, Mauritania, Togo, Niger, Zambia, Chad, Sierra Leone, Central African Republic, Senegal, Tanzania, Cabo Verde, Rwanda, Zimbabwe, and Mozambique. Most of them belong to final clubs with the worst health outcomes. However, countries such as Burundi, Gambia, Madagascar, Namibia, and Eswatini, to mention a few, have increasingly prioritized health spending over time.

However, in most countries, only limited resources have increasingly been allocated to primary and preventive care services, which are critical for substantial progress toward achieving universal health coverage. Moreover, studies have shown limited access to immunization services due to inadequate public health funding and poor infrastructure [28]. This is true for most countries belonging to the final clubs with the worst health outcomes, such as Nigeria, Congo Republic, DRC, and Benin. According to [34] public health spending is crucial in advancing universal health coverage and ensuring equitable access to immunization coverage, which are critical for health equity.

For robustness check, we use the multinomial regression models. The results show the differences in the signs, magnitudes, and significance for all the variables included for the infant and under-five mortality rate models. However, the results are similar regarding the life expectancy at birth model. The multinomial logistic regression results are reported in Table 5 in Appendix 2.

## Conclusion and policy recommendation

Health outcome disparities have become a major concern. Over the years, several studies have attempted to assess the determinants of health inequalities. As part of the Sustainable Development Goals, reducing health outcome disparities is critical, especially for policymakers, given the socioeconomic burdens of such disparities, particularly in Africa. In formulating health policies to address the observed differences, it is crucial to understand the convergence patterns among African countries because countries that converge in the same steady state can adopt common policies more efficiently to the threats to socioeconomic development arising from the pervasive disparities in health outcomes in Africa. Consequently, this study examines the convergence of three health outcomes in 40 African countries from 2000 to 2019. The health outcome proxies include infant mortality rate, under-five mortality rate, and life expectancy at birth. We used a nonlinear time-varying factor model.

Our results provide evidence against the null hypothesis of overall convergence. Instead, we identified the presence of convergence clubs for the three variables of interest. The findings suggest that between-country health outcome disparities have increased over time. Moreover, the results show substantial gaps between the estimated clubs. Regarding infant and under-five mortality rates, final clubs 1, 2, and 3 had the worst outcomes, with infant and under-five mortality rates above 65 deaths per live birth. The results reveal that members of these three final clubs have made little effort to meet the global target of 25 death per 1,000 live births and, thus, catch up with the best-performing clubs. Life expectancies at birth remain low, with a nearly five-year gap between the two final clubs. The study also broadens the literature further by investigating the factors that drive the identified convergence club formation in the selected African countries. The ordered logit models estimated for this purpose suggest economic situations, population structure, urbanization, access to basic sanitation, trade, and external resources for health are significant determinants of club memberships in Africa. Other key variables not assessed in previous studies include DTP immunization coverage among children 12–23 months and public health spending. Moreover, countries with increased immunization and public health expenditure are likelier to belong to convergence clubs with relatively better health outcomes. In contrast, adverse effects are recorded for the convergence clubs with the worst health outcomes for the three models.

Important policy implications can be highlighted based on the results of this study. First, we established that infant mortality rate, under-five mortality rate, and life expectancy at birth in the selected 40 countries converged into seven, six, and two final clubs, respectively. Developing club-specific health policies would help effectively reduce inter-country health disparities in Africa. For example, countries with the highest infant and under-five mortality rates are concentrated in final clubs 1, 2, and 3. For countries in these final clubs, applying more rigorous health policies is advocated, with a significant increase in their national budgets to health as stipulated in the 2001 Abuja declaration and their immunization coverage, access to basic sanitation, and trade. While increasing public health spending is necessary for improved health outcomes, club-specific targeted interventions, such as primary and preventive care services, should be encouraged to meet universal health coverage, thereby reducing disparities in health outcomes. Furthermore, club membership should be considered when developing and negotiating regional agreements in the African Union summits to tackle the persistent health outcome disparities efficiently.

This study has some limitations, which can be addressed in future research. One of the most important limitations is that did not include some important control variables, such as the level of education, and we did not control for important disease burdens, as shown in the literature. Although our study attempted to fill some gaps in the literature on health outcome convergence, there is still room for improvement in future research. Future studies might find it essential to investigate the role of education and disease burdens on health outcome convergence or examine whether spatial interdependence effects matter when analyzing the determinants of health outcome convergence.

## Data Availability

The datasets used in the current study are publicly available and can be found in the World Bank Databank website.

## Appendix 1

List of variables and countries.

**Table:**

**Panel A: List of variables and description**				
Variables	Description		Sources	
**Health expenditure variables**				
U5M	Mortality rate, under-5 (per 1,000 live births)		WDI database	
IMR	Mortality rate, infant (per 1,000 live births)		WDI database	
LE	Life expectancy at birth, total (years)		WDI database	
**Explanatory variables**				
UBPOP	Urban population (% of total population)		WDI database	
POPL15	Population ages 0–14 (% of total population)		WDI database	
POPG65	Population ages 65 and above (% of total population)		WDI database	
EXHE	External health expenditure per capita, PPP (current international $)		WDI database	
RGDp	GDP per capita, PPP (constant 2017 international $)		WDI database	
TO	Trade (% of GDP)		WDI database	
IDTP	DPT immunization (% of children ages 12–23 months)		WDI database	
IMEAS	Immunization, measles (% of children ages 12–23 months)		WDI database	
BASS	People using at least basic sanitation service (% of population)		WDI database	
DGGHE	Domestic general government health expenditure (% of general government expenditure)		WDI database	
**Panel B: List countries**				
Algeria		Guinea		Togo
Angola		Guinea-Bissau		Tunisia
Benin		Kenya		Uganda
Botswana		Madagascar		Zambia
Burkina Faso		Mali		
Burundi		Mauritania		
Cameroon		Mauritius		
Cabo Verde		Morocco		
Central Afr. Rep		Namibia		
Chad		Niger		
Comoros		Nigeria		
Congo, Dem. Rep		Rwanda		
Congo, Rep		Senegal		
Cote d'Ivoire		Sierra Leone		
Equatorial Guinea		South Africa		
Gabon		Sudan		
The Gambia		Swaziland		

WDI represents World Bank's World Development Indicators. Countries were selected on the basis of data availability. We selected 40 African countries based on data availability. The variables were selected based on the existing literature

## Appendix 2

Marginal effects from the multinomial logistic regression results.

**Table 5:** Marginal effects results

Variables	Clubs	Infant mortality rate	Under-5 mortality rate	Life expectancy at birth
Log real GDP per capita	Final club 1	-0.093^a^ (0.018)	-0.083^a^ (0.021)	0.068^b^ (0.032)
	Final club 2	-0.068^a^ (0.011)	0.044 (0.033)	-0.068^b^ (0.032)
	Final club 3	0.075^c^ (0.041)	0.040 (0.029)	
	Final club 4	0.064^c^ (0.034)	-0.029 (0.030)	
	Final club 5	0.321^a^ (0.044)	0.029 (0.032)	
	Final club 6	-0.000^a^ (0.000)	-0.000^b^ (0.000)	
	Final club 7	-0.000^b^ (0.000)		
	Divergent gr	-0.064^b^ (0.020)		
Log population below 15 year old	Final club 1	-1.099^a^ (0.173)	-0.428^b^ (0.210)	0.666^b^ (0.247)
	Final club 2	1.052^a^ (0.185)	1.039^a^ (0.319)	-0.666^b^ (0.247)
	Final club 3	-1.250^a^ (0.336)	0.1221 (0.333)	
	Final club 4	1.220^a^ (0.251)	0.250 (0.243)	
	Final club 5	-0.217 (0.254)	-0.983^a^ (0.299)	
	Final club 6	0.095 (0.076)	-0.000^b^ (0.000)	
	Final club 7	0.000 (0.000)		
	Divergent gr	0.200 (0.299)		
Log population above 65 year old	Final club 1	-0.190^a^ (0.055)	0.148^b^ (0.071)	0.502^a^ (0.107)
	Final club 2	0.472 (0.050)	0.419^a^ (0.107)	-0.502^a^ (0.107)
	Final club 3	-0.156^c^ (0.091)	-0.184^b^ (0.092)	
	Final club 4	0.170^b^ (0.077)	-0.036 (0.079)	
	Final club 5	-0.447^a^ (0.075)	-0.346^b^ (0.139)	
	Final club 6	0.557^a^ (0.079)	0.000^b^ (0.000)	
	Final club 7	0.000^b^ (0.000)		
	Divergent gr	-0.405^a^ (0.063)		
Log urban population	Final club 1	0.175^a^ (0.033)	0.035 (0.030)	-0.427^a^ (0.054)
	Final club 2	0.140^a^ (0.040)	-0.007 (0.038)	0.427^a^ (0.054)
	Final club 3	-0.250^a^ (0.059)	0.052 (0.042)	
	Final club 4	-0.151^a^ (0.037)	0.031 (0.037)	
	Final club 5	-0.144^a^ (0.032)	-0.110^a^ (0.031)	
	Final club 6	0.096^a^ (0.020)	0.000^b^ (0.000)	
	Final club 7	0.000^b^ (0.000)		
	Divergent gr	0.135^b^ (0.052)		
Log external health expenditure	Final club 1	0.324^b^ (0.133)	0.543^b^ (0.259)	-0.253 (0.256)
	Final club 2	0.027 (0.136)	0.330 (0.402)	0.253 (0.256)
	Final club 3	-0.308 (0.443)	-0.696^b^ (0.303)	
	Final club 4	-0.087 (0.243)	0.135 (0.212)	
	Final club 5	0.002 (0.190)	-0.312 (0.253)	
	Final club 6	-0.033 (0.112)	-0.000^b^ (0.000)	
	Final club 7	-0.000 (0.000)		
	Divergent gr	0.077 (0.052)		
Log trade	Final club 1	-0.087^a^ (0 0.013)	-0.040 (0.024)	0.146^a^ (0.036)
	Final club 2	0.003 (0.019)	0.019 (0.033)	-0.146^a^ (0.036)
	Final club 3	0.075^b^ (0.036)	-0.184^a^ (0.032)	
	Final club 4	-0.027 (0.020)	0.183^a^ (0.032)	
	Final club 5	-0.080^a^ (0.015)	0.021 (0.031)	
	Final club 6	0.024^c^ (0.013)	-0.000^b^ (0.000)	
	Final club 7	0.000^b^ (0.000)		
	Divergent gr	0.093^a^ (0.016)		
Log people using at least basic sanitation service	Final club 1	-0.031^c^ (0.018)	-0.011 (0.015)	0.142^a^ (0.033)
	Final club 2	-0.077^a^ (0.015)	-0.019 (0.025)	-0.142^a^ (0.033)
	Final club 3	0.072^c^ (0.043)	-0.072^a^ (0.022)	
	Final club 4	-0.099^b^ (0.039)	-0.004 (0.023)	
	Final club 5	-0.217^a^ (0.027)	0.105^a^ (0.027)	
	Final club 6	0.442^a^ (0.069)	-0.000^b^ (0.000)	
	Final club 7	0.000^b^ (0.000)		
	Divergent gr	-0.092^a^ (0.023)		
Log measles immunization	Final club 1	0.098^b^ (0.048)	-0.037 (0.053)	0.045 (0.140)
	Final club 2	-0.092^b^ (0.037)	-0.006 (0.099)	-0.045 (0.140)
	Final club 3	-0.094 (0.159)	-0.280^b^ (0.116)	
	Final club 4	0.059 (0.107)	0.455^a^ (0.113)	
	Final club 5	0.349^b^ (0.133)	-0.131 (0.130)	
	Final club 6	-0.283^a^ (0.063)	-0.000 (0.000)	
	Final club 7	0.000^b^ (0.000)		
	Divergent gr	-0.036^c^ (0.019)		
Log DTP immunization	Final club 1	-0.326^a^ (0.048)	-0.307^a^ (0.053)	0.217* (0.129)
	Final club 2	0.059 (0.038)	0.038 (0.111)	-0.217* (0.129)
	Final club 3	0.082 (0.163)	0.093 (0.116)	
	Final club 4	0.228^c^ (0.120)	0.331^b^ (0.121)	
	Final club 5	-0.168 (0.130)	-0.156 (0.148)	
	Final club 6	0.175^b^ (0.055)	0.000 (0.000)	
	Final club 7	0.000 (0.000)		
	Divergent gr	-0.050^b^ (0.025)		
Log domestic general government health expenditure	Final club 1	-0.005 (0.009)	-0.040^b^ (0.015)	0.170^a^ (0.032)
	Final club 2	-0.099^a^ (0.010)	-0.180^a^ (0.021)	-0.170^a^ (0.032)
	Final club 3	-0.188^a^ (0.027)	-0.064^b^ (0.025)	
	Final club 4	0.294^a^ (0.026)	0.313^a^ (0.028)	
	Final club 5	0.029 (0.019)	-0.030 (0.026)	
	Final club 6	0.006 (0.009)	-0.000^b^ (0.000)	
	Final club 7	-0.000 (0.000)		
	Divergent gr	-0.037^a^ (0.010)		
**No. of observation**		**787**	**787**	**787**
***P*** **-Values**		**0.0000**	**0.0000**	**0.0000**

^a^, ^b^, and ^c^ represent statistical significance at 1%, 5%, and 10%, respectively. The standard errors are in parenthesis. The first column present the variables’ code, while the second one shows the final clubs. The third column presents the marginal effects results for the infant mortality rate model, the fourth column provides the results of the under-five mortality rate model, and the fifth column provides the results for the life expectancy model. Source: Authors’ own computations
